# Effects of luteolin on proliferation and apoptosis of breast cancer cells by regulating EGFR/STAT3/AKt pathway

**DOI:** 10.1515/biol-2025-1270

**Published:** 2026-05-18

**Authors:** Xueyu Zheng, Pinting Fu, Zhihua Xia, Wen Feng, Bingbao Song

**Affiliations:** Department of Thyroid and Breast Surgery, Department of General Surgery, Putuo District Central Hospital, Shanghai, 200000, China

**Keywords:** LU, BCCs, proliferative capacity, AR, EGFR

## Abstract

Breast cancer (BC) is a common malignant tumor in women, characterized by high recurrence rates, a significant risk of metastasis, and limitations of existing treatments. The anticancer activity of the natural compound luteolin (LU) has been confirmed, but its specific mechanism of action in breast cancer cells (BCCs) has not been fully elucidated. This study aimed to investigate the effects of LU on the proliferation and apoptosis of BCCs and to clarify its association with the epidermal growth factor receptor (EGFR)/signal transducer and activator of transcription 3 (STAT3)/protein kinase B (Akt) pathway. Using MCF-7 breast cancer cells as the research subject, experimental groups were set up with 5, 15, 25, 35, and 45 μmol/L LU treatment and a dimethyl sulfoxide (DMSO) solvent control group. Cell proliferation activity, reactive oxygen species (ROS) levels, apoptosis rate (AR), and the expression of phosphorylated Akt (p-Akt), phosphorylated EGFR (p-EGFR), and phosphorylated STAT3 (p-STAT3) were detected. LU at concentrations of 25, 35, and 45 μmol/L significantly inhibited the proliferation of MCF-7 cells (*P* < 0.05), while increasing the cell apoptosis rate and ROS levels (*P *<* *0.05). Within this concentration range, the expression of p-EGFR, p-STAT3, and p-Akt was significantly downregulated (*P *<* *0.05), whereas the total protein expression levels of EGFR, Akt, and STAT3 showed no significant change (*P *>* *0.05). The inhibitory effect of LU on MCF-7 cell proliferation and its pro-apoptotic effect were dose-dependent, with half-maximal inhibitory concentrations (IC_50_) of 28.7 ± 1.5 μmol/L and 29.4 ± 1.8 μmol/L, respectively. LU dose-dependently inhibits the proliferation and promotes the apoptosis of MCF-7 cells by specifically inhibiting the phosphorylation activation of the EGFR/STAT3/Akt pathway and inducing ROS accumulation. This provides experimental evidence for LU as a potential therapeutic agent for breast cancer.

## 1 Introduction

Breast cancer (BC) is recognized as one of the most prevalent malignant neoplasms among women globally. Its incidence and mortality remain high, which seriously threatens women’s health [1], 2]. Despite notable advancements in the early detection and therapeutic strategies for BC, the issues of high recurrence and metastatic potential persist, presenting ongoing challenges in clinical management. The treatment difficulty of BC is mainly reflected in the following aspects: the heterogeneity of tumor leads to inconsistent treatment response, drug resistance makes some patients no longer sensitive to existing therapies, and the invasiveness and metastasis of tumor cells increase the complexity of treatment [3], [4], [5]. In addition, there are still some difficulties in early screening and diagnosis of BC, especially for some subtypes of BC, which lack specific markers [6]. Currently, the primary therapeutic approaches for BC encompass surgical excision, radiation therapy, chemotherapy, hormonal therapy, and targeted therapy. Surgical resection is the main treatment of early BC, which can control the disease by removing the tumor tissue. Radiotherapy kills cancer cells with high-energy rays, usually in combination with surgery or chemotherapy. Chemotherapy uses drugs to kill or inhibit the growth of cancer cells, which is suitable for advanced or metastatic BC. Endocrine therapy targets hormone receptor-positive BC, aiming to curb malignant cells by interfering with the action of hormones [7], 8]. Targeted therapy is a new type of therapy developed in recent years, which has high specificity and fewer side effects by targeting specific molecular pathways or gene mutations [9], 10]. However, there are still various limitations and challenges in the practical application of these therapeutic methods, and there exists an immediate necessity to discover innovative treatment modalities and pharmaceutical agents.

In recent years, more and more studies have focused on the potential of natural compounds in cancer treatment. Luteolin (LU), as a flavonoid, has received extensive attention due to its remarkable anticancer properties [11]. LU widely exists in a variety of plants and can suppress the proliferation of cancer cells and induce apoptosis through a variety of pathways [12], 13]. Jiang et al. (2021) [14] proposed that LU exhibited potent anticancer activity *in vivo* in H358 xenograft and Lewis lung cancer models, significantly suppressing the proliferation of lung cancers with mutated murine sarcoma virus cancer genes, which might be a prospective therapeutic strategy for Kirsten rat sarcoma 2 viral oncogene homolog (KRAS)-mutant non-small cell lung cancer (NSCLC). Zheng et al. [15] found that the combined treatment of LU and Erlotinib inhibited the viability and proliferation of colon cancer cells by regulating the decreased expression of glutathione peroxidase 4 (GPX4) mediated by cancer1 gene. However, significant research gaps remain: current studies on the anticancer effects of LU have primarily focused on malignancies such as lung and colorectal cancers, while its specific mechanisms of action in BC, particularly its regulatory effects on key signaling pathways driving BC progression, have not been fully elucidated. However, the specific mechanism of LU in BC has not been fully elucidated. Existing studies have shown that epidermal growth factor receptor (EGFR) and its downstream signal transducer and activator of transcription 3 (STAT3), and protein kinase B (Akt) signaling pathways are pivotal in the growth and survival of breast cancer cells (BCCs) [16], [17], [18]. Numerous studies have confirmed the potential value of LU in breast cancer treatment, but existing evidence reveals a significant imbalance in subtype-specific research. Current investigations into the anti-breast cancer mechanisms of LU have predominantly focused on the highly aggressive triple-negative breast cancer (TNBC) subtype. For instance, Wu et al. [19] demonstrated that LU can simultaneously induce apoptosis and autophagy in TNBC cells by regulating the SGK1-FOXO3a-BNIP3 signaling pathway, thereby exerting growth-inhibitory effects. Wang et al. [20] found that the combined application of LU and curcumin significantly enhances the suppression of TNBC cell proliferation, colony formation, and metastasis by synergistically activating the type I interferon signaling pathway and inhibiting the transforming growth factor-β (TGF-β) signaling pathway. However, these mechanisms identified in TNBC cannot be directly extrapolated to the hormone receptor-positive breast cancer subtype, which accounts for a larger proportion of clinical breast cancer cases. The pathogenesis, signaling pathway characteristics, and treatment dependencies (such as sensitivity to hormonal therapies) of this subtype fundamentally differ from those of TNBC. Consequently, the specific mechanisms of action of LU in this subtype have long remained an understudied area. This study, using MCF-7 cells, investigated the effects of different concentrations of LU on EGFR/STAT3/Akt pathway phosphorylation levels, cell proliferation, and apoptosis, and quantifies the half-maximal inhibitory concentration (IC50) values for proliferation inhibition and apoptosis induction. It thereby addressed the gap in LU research for hormone receptor-positive BC and, through correlation analysis of dose-effect-pathway, overcomes the limitations of previous studies, such as insufficient mechanistic depth and lack of quantitative data.

It remains unclear whether LU can target the EGFR/STAT3/Akt pathway in BC cells, and if so, whether it regulates cellular biological behavior by modulating the phosphorylation activation of these proteins rather than their total protein expression. This constitutes a key research gap that the present study aims to address. Based on the above, this study proposed the following hypothesis: LU specifically inhibits the phosphorylation of EGFR, STAT3, and Akt without affecting their total protein expression, thereby blocking the activation of the EGFR/STAT3/Akt signaling pathway, which in turn inhibits proliferation and promotes apoptosis in MCF-7 BC cells. Additionally, LU may induce intracellular reactive oxygen species (ROS) accumulation by inhibiting this pathway, further enhancing its pro-apoptotic effect.

## 2 Materials and methods

### 2.1 Cell line

MCF-7 cells were purchased from Shanghai Fuheng Biotechnology Co., Ltd. The cells were authenticated by short tandem repeat (STR) profiling (performed by Shanghai Boying Biotechnology Co., Ltd.), showing a 100 % match with the standard MCF-7 cell line (ATCC^®^ HTB-22™). Prior to inoculation and every three passages during culture, mycoplasma contamination was tested using the MycoAlert™ Mycoplasma Detection Kit (Lonza, Switzerland), and all results were negative. The number of cells used in the experiments was maintained between 15 and 25 to ensure phenotypic and functional stability.

The human breast cancer MCF-7 cell line was selected as the experimental model in this study for the following reasons: First, MCF-7 cells are a classic model for hormone receptor-positive breast cancer, a subtype that represents a high proportion of clinical breast cancer cases. Using this model enhances the clinical relevance and translational potential of the research findings. Second, the EGFR/STAT3/AKT pathway is abnormally activated in MCF-7 cells, which aligns with the core objective of this study – focusing on the targeted regulation of this pathway by LU – allowing for precise validation of LU’s regulatory effects. Additionally, MCF-7 cells exhibit stable growth and consistent phenotypes *in vitro*, enabling clear observation of dose-dependent therapeutic effects and providing a reliable basis for analyzing cellular responses, thereby ensuring the reproducibility of the experiments.

### 2.2 Cell culture

The culture and subculture protocol for MCF-7 cells is as follows: Complete medium, consisting of Dulbecco’s Modified Eagle Medium (DMEM, Thermo Fisher Scientific) supplemented with 10 % fetal bovine serum (FBS; Gibco, Cat. No. 10099141C) and 1 % penicillin-streptomycin (Thermo Fisher Scientific), was pre-warmed to 37 °C before use. For cryopreserved cell resuscitation, the cryovial was quickly thawed in a 37 °C water bath (within 2 min) and then transferred into a centrifuge tube containing 5 mL of pre-warmed complete medium, followed by centrifugation at 1,000×*g* for 5 min to remove the cryoprotectant. The cell suspension was seeded into a T25 culture flask (Corning, USA) and incubated at 37 °C in a humidified atmosphere with 5 % CO_2_ and 95 % relative humidity.

When the cells reached 70–80 % confluency, subculture was performed. The cells were washed twice with phosphate-buffered saline (PBS; Thermo Fisher Scientific, Cat. No. 10010023) and then treated with 0.25 % trypsin-ethylenediaminetetraacetic acid (EDTA) solution (Shanghai Jike Biotechnology Co., Ltd., Cat. No. C0201) at 37 °C for 1–2 min. Once cell detachment was observed under an inverted microscope, an equal volume of complete medium was added to stop the digestion. After centrifugation at 1,000×*g* for 5 min and removal of the supernatant, the cells were resuspended in fresh complete medium and seeded into new T25 flasks at a 1:3 split ratio, followed by further incubation. The medium was routinely changed every 2–3 days, and cell growth and condition were monitored daily using an inverted microscope to ensure cell health and timely subculture.

### 2.3 Drug intervention method

LU (LU, Shanghai Yuanye Bio-Technology Co., Ltd., purity ≥98 %) was dissolved in dimethyl sulfoxide (DMSO, Hunan Xiangmao Pharmaceutical & Chemical Co., Ltd., purity ≥99.9 %, Product No.: XM-0102) to prepare a 50 mmol/L stock solution. After filtering through a 0.22 µm sterile filter (Millipore, USA) to remove insoluble impurities, the solution was aliquoted into sterile centrifuge tubes and stored at −20 °C protected from light and high temperature. Before use, the stock solution was thawed at room temperature and diluted with complete medium to the required working concentrations.

The prepared LU working solutions were added to the MCF-7 cell culture system and incubated at 37 °C with 5 % CO_2_ for 24 h. The concentration gradients used were 5, 15, 25, 35, and 45 μmol/L. In all treatment groups and the solvent control group, the final concentration of DMSO was ≤0.1 % (v/v). Preliminary experiments confirmed that this concentration had no significant effect on the proliferation, apoptosis, or pathway protein expression of MCF-7 cells.

A positive control group was established: referring to the selection method for positive controls in breast cancer cells from Reference [19], doxorubicin (Pfizer Pharmaceuticals Ltd., Wu xi) was added at a final concentration of 10 μmol/L. A solvent control group was established: an equal volume of DMSO (final concentration 0.1 %) was added. All treatment groups, positive control group, and solvent control group were set up with three technical replicates, and the experiment was independently repeated three times (technical replicates represent intra-experiment repeats, while independent experiments represent biological replicates).

### 2.4 Western blotting (WB)

WB was used to detect the expression of EGFR and p-EGFR, STAT3 and p-STAT3, Akt and p-Akt in MCF-7. The specific steps: MCF-7 was rinsed with PBS, digested with trypsin, centrifuged, and the cell pellet was collected. The cell suspension was applied to cold RIPA lysis buffer (Thermo Fisher Scientific), incubation for 15–30 min to lyse the cells, and the suspension was pipetted to ensure complete lysis. After centrifugation to remove cell debris, the upper liquid was collected, and protein concentration was measured. The proteins were adjusted to the same concentration for analysis. Then, electrophoresis was conducted to fractionate the proteins, and subsequently, they were transferred onto a nitrocellulose membrane (Membrane Solutions Co., Ltd.) using Tris-glycine buffer (Sangon Biotech (Shanghai) Co., Ltd.), set at 100 V for 1 h. After transfer, the membrane was blocked with 5 % skim milk for 1 h to prevent non-specific binding. Then, specific first antibodies (Abs) against EGFR, p-EGFR, JAK2, p-JAK2, STAT3, p-STAT3, Akt, and p-Akt were diluted based on the guidance, incubation overnight at 4° C. Following rinsing the membrane to remove unbound first Ab, horseradish peroxidase (HRP) labeled secondary Ab were diluted, incubation at 25 °C for 1 h. The membrane was rinsed again to remove unbound secondary Ab. Finally, the proteins were visualized using an enhanced chemiluminescent (ECL) kit (Vazyme Biotech Co., Ltd., Nanjing), the membrane was exposed to an X-ray film and developed to obtain the protein band images. The bands on the membrane were analyzed using imaging analysis software to determine the relative expression, which was normalized using glyceraldehyde-3-phosphate dehydrogenase (GAPDH). The experiment was independently repeated three times, with each group set up in triplicate (biological replicates *n *=* *3, each containing three technical replicates).

All Abs used in the WB analysis were validated commercial Abs with high specificity. The details were as follows: rabbit anti-human phospho-Akt (p-Akt, Ser473) polyclonal Ab, rabbit anti-human total Akt polyclonal Ab, rabbit anti-human phospho-EGFR (p-EGFR, Tyr1068) polyclonal Ab, rabbit anti-human total EGFR polyclonal Ab, rabbit anti-human phospho-STAT3 (p-STAT3, Tyr705) polyclonal Ab, rabbit anti-human total STAT3 polyclonal Ab, and mouse anti-human β-actin monoclonal Ab (loading control). The secondary Abs used were HRP-conjugated goat anti-rabbit IgG and goat anti-mouse IgG. All Abs were diluted according to the manufacturer’s recommended ratios to ensure the specificity and reliability of the detection results.

### 2.5 Proliferative activity

After the treatment time ended, 10 µL of 5 mg/mL 3-(4,5-Dimethylthiazol-2-yl)-2,5-Diphenyltetrazolium Bromide (MTT) solution (Shanghai Mao Kang Biotechnology Co., Ltd.) was applied, continuing to incubate at 37 °C, 5 % CO_2_ for 4 h, allowing MTT to enter the cells and be converted into purple formazan crystals. Following the incubation ended, 150 µL of DMSO (Thermo Fisher Scientific) was applied, and the plate was gently shaken to facilitate the dissolution of the formazan crystals. Finally, the optical density (OD) of each well was measured at a wavelength of 570 nm using a Multiskan SkyHigh microplate reader (Thermo Fisher Scientific). The proliferative rate was calculated based on the OD value, and the proliferative inhibition rate or proliferative rate was determined by comparing the OD values of the treated groups with those of the control group (CG). The experiment was independently repeated 3 times, with three replicate wells set per group (*n* = 3).

The CCK-8 assay followed the same experimental grouping as above, with three replicate wells per group and three independent repetitions. MCF-7 cells were seeded at 5 × 10^3^ cells per well in a 96-well plate. After adherence, corresponding reagents were added and incubated. At each time point, 10 μL of cell counting kit-8 (CCK-8) reagent (Beyotime Biotechnology, Shanghai) was added to each well, followed by incubation at 37 °C for 1 h. The OD value was measured at 450 nm using a microplate reader, and proliferation activity was calculated. The experiment was independently repeated three times with three replicate wells per group (*n* = 3).

A colony formation experiment was conducted with three replicate wells per group and independently repeated three times. MCF-7 cells were seeded at 500 cells per well in 6-well plates. After adherence, corresponding reagents were added, and the cells were cultured at 37 °C with 5 % CO_2_ for 14 days (with medium change every 3 days). After cultivation, the medium was discarded, and the cells were washed twice with PBS. They were fixed with 4 % paraformaldehyde for 30 min and stained with 0.1 % crystal violet for 15 min, followed by rinsing with running water and air-drying. Colonies containing ≥50 cells were counted under an inverted microscope, and the colony formation rate was calculated [Colony formation rate = (number of colonies in experimental group/number of colonies in solvent CG) × 100  %]. The experiment was independently repeated three times, with three replicate wells per group (*n* = 3).

### 2.6 Apoptosis rate (AR)

The apoptosis of MCF-7 was detected using the Hoechst staining method. First, MCF-7 was processed into a single cell suspension and added to a culture dish on a glass slide. Then, staining was carried out using the Hoechst dye at 1 μg/mL, incubation at 37 °C for 20 min. Finally, the stained MCF-7 was subjected to observation using a high-definition fluorescence microscope (Maxidyne (Dongguan) Technology Co., Ltd.), and the AR was computed. The experiment was independently repeated three times with three replicate wells per group (*n* = 3).

For the Hoechst 33258 staining assay to detect apoptosis, after MCF-7 cells were treated with different concentrations of LU, the culture medium was discarded, and the cells were gently washed twice with pre-cooled PBS. Then, 5 μg/mL Hoechst 33258 staining solution was added to each well, and the cells were incubated at 37 °C in a 5 % CO_2_ incubator for 15 min in the dark. After incubation, the cells were washed three times with PBS to remove residual staining solution and observed under a fluorescence microscope. For quantification, five non-overlapping high-power fields (200×) were randomly selected per sample slide, with at least 200 cells counted per field. The percentage of cells exhibiting dense, bright blue nuclei (characteristic of apoptotic cells) relative to the total number of cells was calculated. To minimize observer bias, the entire procedure was conducted in a double-blind manner: two researchers not involved in the experimental grouping independently performed field selection, cell counting, and data recording. The average of their results was taken as the final AR for each sample to ensure objectivity and reliability.

### 2.7 Detection of ROS levels

First, well-grown MCF-7 was distributed into a 96-well plate, and based on the guidance of the ROS detection kit (Beijing Biolab Technology Co., Ltd.), 1 mL of dye was applied, incubation for 20 min. Following rinsing three times with phenol red-free 1640 medium, photos were taken, and the OD value was subjected to measurement. The experiment was independently repeated three times, with three replicates per group (*n* = 3).

The procedure was performed according to the kit instructions: 1 mL of diluted dye working solution was added to each well (the dye was pre-warmed to room temperature and briefly centrifuged to ensure the solution was concentrated at the bottom of the tube). The cells were incubated at 37 °C in a 5 % CO_2_ incubator for 20 min in the dark (all steps were carried out under light-protected conditions to avoid dye oxidation and false positives). After incubation, the cells were gently washed three times with phenol red-free 1640 medium. After each wash, the supernatant was completely removed to eliminate residual extracellular dye and reduce background fluorescence interference. Images were then captured under a fluorescence microscope, and the fluorescence intensity of each well was measured using a fluorescence plate reader (detection parameters were set as recommended by the kit, *e.g.,* excitation wavelength 488 nm, emission wavelength 525 nm; fluorescence intensity was uniformly expressed in relative fluorescence units instead of OD values). All measurements were performed with three replicate wells, and the average values were used for statistical analysis to ensure reliability and reproducibility of the results.

### 2.8 Dose-response analysis and kinetic parameter fitting

To systematically analyze the concentration-dependent effects of LU on MCF-7 cells, dose-response curves and kinetic parameter fitting were performed using GraphPad Prism 9.0 software. The specific steps are as follows: curve plotting: LU concentrations (5, 15, 25, 35, and 45 μmol/L) were plotted on the *x*-axis, with cell proliferation inhibition rate, AR, ROS levels, and protein phosphorylation levels on the *y*-axis. A nonlinear regression model (Logistic function) was used to fit the curve, with the equation: $Y=\frac{\text{B}\text{o}\text{t}\text{t}\text{o}\text{m}+\left(\text{T}\text{o}\text{p}‐\text{B}\text{o}\text{t}\text{t}\text{o}\text{m}\right)}{1+10\left(\text{L}\text{o}\text{g}\text{E}\text{C}50‐\text{X}\right)\times \text{H}\text{i}\text{l}\text{l}\text{S}\text{l}\text{o}\text{p}\text{e}\text{B}\text{o}\text{t}\text{t}\text{o}\text{m}}$ .

Where *X* represents the drug concentration, *Y* is the response value, Bottom and Top denote the minimum and maximum response values, LogEC50 is the logarithm of the concentration yielding half-maximal effect, and HillSlope is the slope factor. Parameter extraction was performed. The IC50, Hill coefficient, and maximal effect value (Emax) were calculated. The reliability of the parameters was verified through 95 % confidence intervals (CI). Analysis of variance (ANOVA) was used to assess the goodness of curve fitting (*P* < 0.05 was considered significant).

### 2.9 ROS scavenging assay

Four experimental groups were set up: solvent control group (complete medium containing 0.1 % DMSO), LU (LU) alone treatment group (45 μmol/L LU, the concentration inducing the highest apoptosis rate), NAC alone treatment group (5 mmol/L N-acetylcysteine, a ROS scavenger; Sigma-Aldrich, Product code: A7250), and LU + NAC co-treatment group (pretreatment with 5 mmol/L NAC for 2 h, followed by incubation with 45 μmol/L LU for 24 h). The apoptosis rate was detected by Hoechst 33258 staining, and ROS levels were measured using a ROS detection kit. Each group had three replicate wells, and the experiment was independently repeated three times (*n* = 3). By comparing the differences in indicators among the groups, the mediating role of ROS accumulation was validated.

### 2.10 Pathway rescue experiment

MCF-7 cells were seeded in 6-well plates. At 60 % confluence, they were transfected with pcDNA3.1-EGFR-CA (constitutively active EGFR plasmid), pcDNA3.1-STAT3-CA (constitutively active STAT3 plasmid), pcDNA3.1-Akt-CA (constitutively active Akt plasmid), or the empty vector pcDNA3.1 (Addgene). Transfection was performed using Lipofectamine 3000 (Thermo Fisher Scientific, Product code: L3000015) according to the manufacturer’s instructions. Twenty-four hours after transfection, cells were treated with 45 μmol/L LU and incubated for an additional 24 h. Three groups were established: empty vector control group (transfected with empty vector only, no LU treatment), empty vector + LU group (transfected with empty vector followed by LU treatment), and CA plasmid + LU group (transfected with the corresponding CA plasmid followed by LU treatment). Apoptosis rate was detected solely by Hoechst 33258 staining. Each group had three replicate wells, and the experiment was independently repeated three times (*n* = 3). By comparing the apoptosis rates among groups, the direct correlation between pathway inhibition and apoptosis was verified.

### 2.11 Validation experiment in different breast cancer subtypes

The triple-negative breast cancer cell line MDA-MB-231 (Shanghai Fuheng Biotechnology Co., Ltd.) was introduced to conduct parallel experiments alongside MCF-7 cells, aiming to validate the universal effect of LU across subtypes.

Cells were cultured in DMEM medium supplemented with 10 % fetal bovine serum and 1 % penicillin-streptomycin, under conditions of 37 °C, 5 % CO_2_, and 95 % relative humidity. The passage number was controlled between 15 and 25, and mycoplasma testing was negative. Similar to MCF-7 cells, groups included solvent control, LU treatment groups (5, 15, 25, 35, 45 μmol/L), and a positive control group (10 μmol/L doxorubicin). Measured indicators: cell proliferation activity (MTT assay) and apoptosis rate (Hoechst 33258 staining) were assessed simultaneously. Each group had three replicate wells, and the experiment was independently repeated three times (*n* = 3).

### 2.12 Statistical processing

Data analysis was carried out utilizing SPSS 22.0. All experimental data were tested for normality using the Shapiro-Wilk test prior to ANOVA analysis. Quantitative data that adhered to a normal distribution were depicted in the format of mean ± SD (*x̄* ± *s*), whereas categorical data were depicted in terms of frequency and percentage (%). Non-normally distributed quantitative data were analyzed using the Mann-Whitney test, and normally distributed quantitative data were analyzed using one-way ANOVA. Categorical data were compared utilizing the chi-square test. The distinction was statistically considerable with *P* < 0.05.

### 2.13 Research ethics

This study was conducted in accordance with the ethical guidelines for in vitro cell experiments. Commercially available human breast cancer cell lines (MCF-7) were used, and no human or animal subjects were involved in this study.

### 2.14 Informed consent

This study does not involve human subjects, patient samples, or personally identifiable information, and therefore does not require informed consent.

## 3 Results

### 3.1 Analysis of cell proliferation activity and clone formation ability

**Figure 1:**
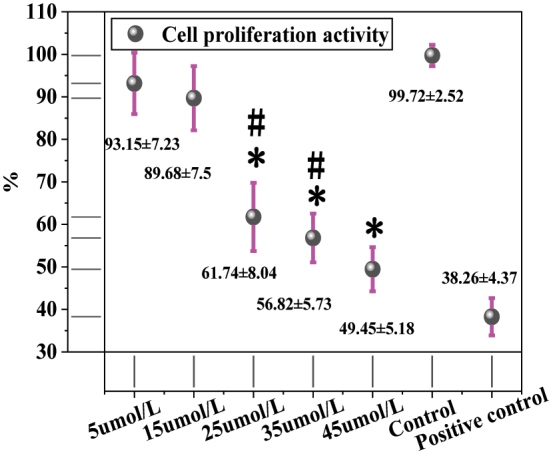
Contrast of cell multiplication activity using LU. * as against the CG, # as against 45 μmol/L LU, *P* < 0.05

Figure 1 shows the proliferation activity of MCF-7 cells after treatment with different concentrations of LU. The cell proliferation activity was 93.15 ± 7.23 % in the 5 μmol/L LU group, 89.68 ± 7.55 % in the 15 μmol/L group, 61.74 ± 8.04 % in the 25 μmol/L group, 56.82 ± 5.73 % in the 35 μmol/L group, 49.45 ± 5.18 % in the 45 μmol/L group, and 99.72 ± 2.52 % in the solvent CG. The positive CG showed a proliferation activity of 38.26 ± 4.37 %. Intergroup comparisons revealed that, compared to the solvent CG, the 25 μmol/L, 35 μmol/L, and 45 μmol/L LU treatment groups exhibited significantly reduced proliferation activity (*P* < 0.05). The positive CG also showed significantly lower proliferation activity than the solvent CG (*P* < 0.01). Further comparison between LU treatment groups and the positive CG showed that the 5 μmol/L, 15 μmol/L, 25 μmol/L, and 35 μmol/L LU groups had significantly higher proliferation activity than the positive CG (*P* < 0.05). Although the 45 μmol/L LU group (49.45 ± 5.18 %) showed higher proliferation activity than the positive CG, the difference was not statistically significant (*P* > 0.05). Additionally, the proliferation activity in the 45 μmol/L LU group was significantly lower than that in the 25 μmol/L and 35 μmol/L groups (*P* < 0.05).

**Figure 2:**
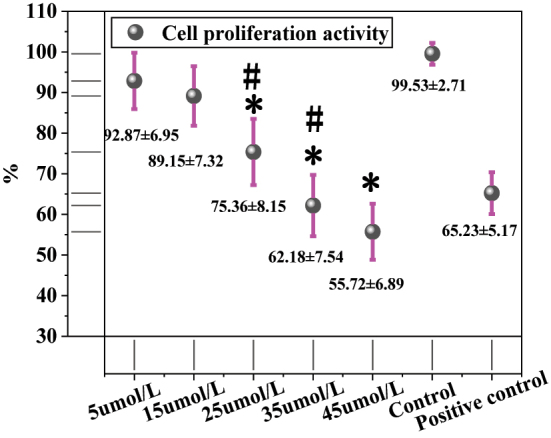
Results of the CCK-8 assay. * as against the CG, # as against 45 μmol/L LU, *P* < 0.05.

Figure 2 presents the results of the CCK-8 assay. The cell proliferation activity was 92.87 ± 6.95 % in the 5 μmol/L LU group, 89.15 ± 7.32 % in the 15 μmol/L group, 75.36 ± 8.15 % in the 25 μmol/L group, 62.18 ± 7.54 % in the 35 μmol/L group, 55.72 ± 6.89 % in the 45 μmol/L group, and 99.53 ± 2.71 % in the solvent CG. The positive CG exhibited a proliferation activity of 65.23 ± 5.17 %. Intergroup comparisons revealed that, compared to the solvent CG, the 25 μmol/L, 35 μmol/L, and 45 μmol/L LU treatment groups showed significantly reduced proliferation activity (*P* < 0.05). The positive CG also demonstrated significantly lower proliferation activity than the solvent CG (*P* < 0.01). Further comparison between the LU treatment groups and the positive CG indicated that the 5 μmol/L, 15 μmol/L, and 25 μmol/L LU groups had significantly higher proliferation activity than the positive CG (*P* < 0.05). Although the proliferation activities of the 35 μmol/L and 45 μmol/L LU groups (62.18 ± 7.54 % and 55.72 ± 6.89 %, respectively) were lower than that of the positive CG, the differences were not statistically significant (*P* > 0.05). Additionally, the proliferation activity in the 45 μmol/L LU group was significantly lower than that in the 25 μmol/L and 35 μmol/L groups (*P* < 0.05).

**Figure 3:**
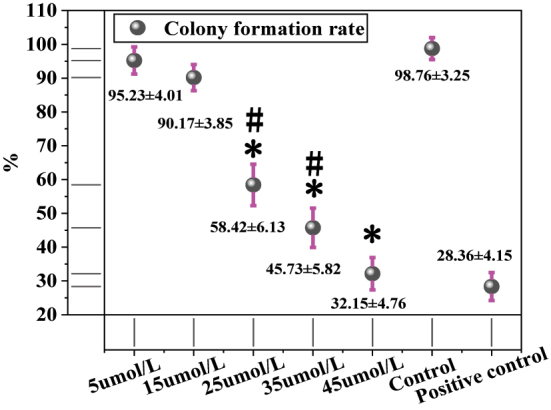
Colony formation of MCF-7 cells after treatment with different concentrations of LU. * as against the CG, # as against 45 μmol/L LU, *P* < 0.05.

Figure 3 shows the colony formation of MCF-7 cells after treatment with different concentrations of LU. The colony formation rate was 95.23 ± 4.01 % in the 5 μmol/L LU group, 90.17 ± 3.85 % in the 15 μmol/L group, 58.42 ± 6.13 % in the 25 μmol/L group, 45.73 ± 5.82 % in the 35 μmol/L group, 32.15 ± 4.76 % in the 45 μmol/L group, and 98.76 ± 3.25 % in the solvent CG. The positive CG exhibited a colony formation rate of 28.36 ± 4.15 %. Intergroup comparisons revealed that, compared to the solvent CG, the 25 μmol/L, 35 μmol/L, and 45 μmol/L LU treatment groups showed significantly reduced colony formation rates (*P* < 0.05). The positive CG also demonstrated a significantly lower colony formation rate than the solvent CG (*P* < 0.01). Further comparison between the LU treatment groups and the positive CG indicated that the 5 μmol/L, 15 μmol/L, 25 μmol/L, and 35 μmol/L LU groups had significantly higher colony formation rates than the positive CG (*P* < 0.05). Although the colony formation rate in the 45 μmol/L LU group (32.15 ± 4.76 %) was higher than that in the positive CG, the difference was not statistically significant (*P* > 0.05). Additionally, the colony formation rate in the 45 μmol/L LU group was significantly lower than that in the 25 μmol/L and 35 μmol/L groups (*P* < 0.05).

### 3.2 Contrast of apoptosis

**Figure 4:**
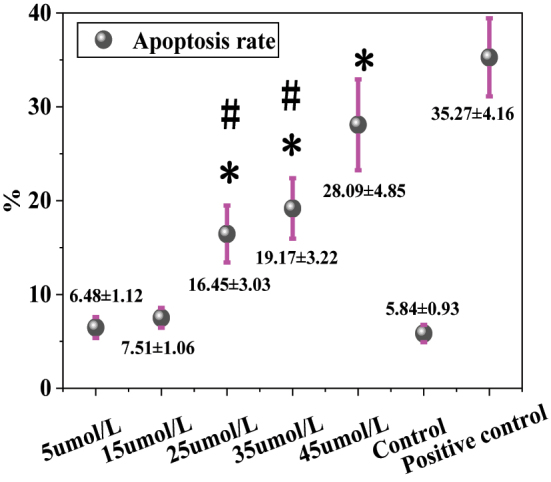
Contrast of AR. * as against the CG, # as against 45 μmol/L LU, *P* < 0.05.

**Figure 5:**
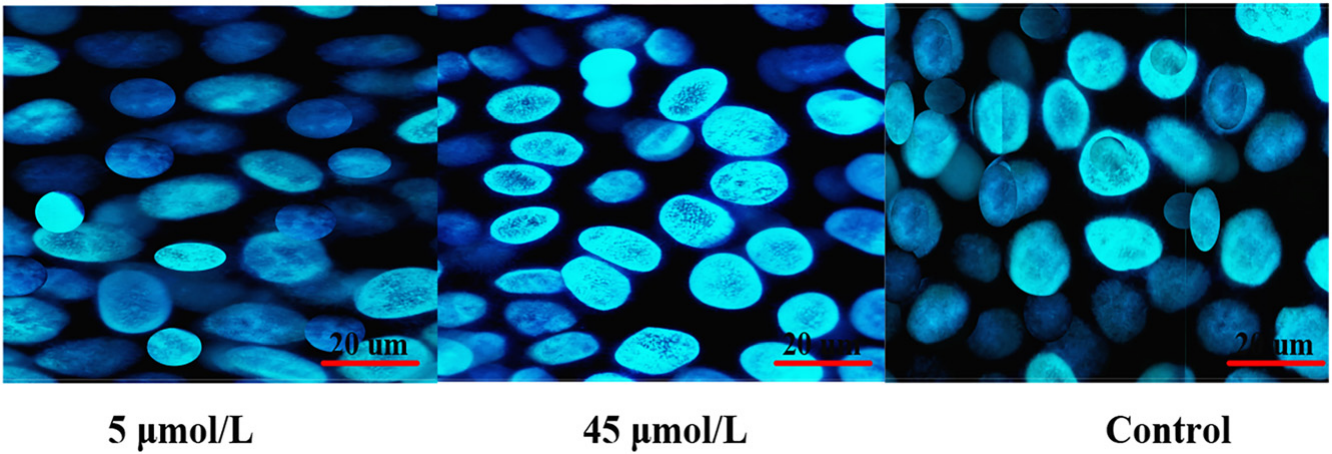
Cell staining images of the 5 μmol/L LU, 45 μmol/L LU groups, and CG.

Figure 4 shows the ARs of MCF-7 cells treated with different concentrations of LU and the positive control. The AR was 6.48 ± 1.12 % in the 5 μmol/L LU group, 7.51 ± 1.06 % in the 15 μmol/L group, 16.45 ± 3.03 % in the 25 μmol/L group, 19.17 ± 3.22 % in the 35 μmol/L group, 28.09 ± 4.85 % in the 45 μmol/L group, and 5.84 ± 0.93 % in the solvent CG. The positive CG exhibited an AR of 35.27 ± 4.16 %. Intergroup comparisons revealed that, compared to the solvent CG, the 25 μmol/L, 35 μmol/L, and 45 μmol/L LU treatment groups showed significantly increased ARs (*P* < 0.05). The positive CG also demonstrated a significantly higher AR than the solvent CG (*P* < 0.01). Further comparison between the LU treatment groups and the positive CG indicated that the 5 μmol/L, 15 μmol/L, 25 μmol/L, and 35 μmol/L LU groups had significantly lower ARs than the positive CG (*P* < 0.05). Although the AR in the 45 μmol/L LU group (28.09 ± 4.85 %) was lower than that in the positive CG, the difference was not statistically significant (*P* > 0.05). Additionally, the AR in the 45 μmol/L LU group was significantly higher than that in the 25 μmol/L and 35 μmol/L groups (*P* < 0.05). Figure 5 shows the staining images of cells in the 5 μmol/L LU, 45 μmol/L LU, and positive CGs. As the LU concentration increased, cell density gradually decreased, and morphological shrinkage was observed.

### 3.3 EGFR and p-EGFRprotein expression

**Figure 6:**
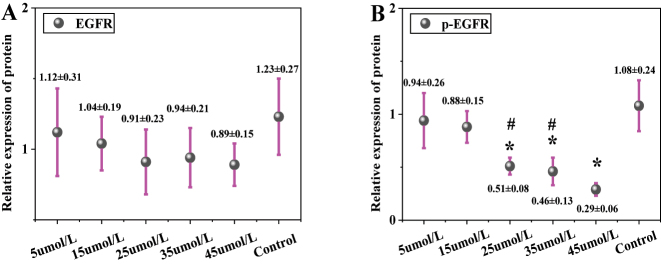
Contrast of EGFR and p-EGFR protein expression (A for EGFR; B for p-EGFR). * as against the CG, # as against 45 μmol/L LU, *P* < 0.05.

In Figure 6A, the total EGFR protein expression was 1.12 ± 0.31 in the 5 μmol/L LU group, 1.04 ± 0.19 in the 15 μmol/L group, 0.91 ± 0.23 in the 25 μmol/L group, 0.94 ± 0.21 in the 35 μmol/L group, 0.89 ± 0.15 in the 45 μmol/L group, and 1.23 ± 0.27 in the CG. No significant differences in total EGFR protein expression were observed between any LU concentration groups (5, 15, 25, 35, 45 μmol/L) and the CG (*P* > 0.05). In Figure 6B, the p-EGFR protein expression was 0.94 ± 0.26 in the 5 μmol/L LU group, 0.88 ± 0.15 in the 15 μmol/L group, 0.51 ± 0.08 in the 25 μmol/L group, 0.46 ± 0.13 in the 35 μmol/L group, 0.29 ± 0.06 in the 45 μmol/L group, and 1.08 ± 0.24 in the CG. The p-EGFR protein expression decreased significantly with increasing LU concentration. Compared to the CG, the 25 μmol/L, 35 μmol/L, and 45 μmol/L LU treatment groups showed significantly downregulated p-EGFR expression (*P* < 0.05). Moreover, the p-EGFR expression in the 45 μmol/L LU group was significantly lower than that in the 25 μmol/L and 35 μmol/L groups (*P* < 0.05). The p-EGFR/EGFR ratio was further calculated. The ratio was 0.84 ± 0.08 in the 5 μmol/L LU group, 0.85 ± 0.06 in the 15 μmol/L group, 0.56 ± 0.05 in the 25 μmol/L group, 0.49 ± 0.04 in the 35 μmol/L group, 0.33 ± 0.03 in the 45 μmol/L group, and 0.88 ± 0.07 in the CG. These results indicate that the proportion of p-EGFR relative to total EGFR gradually decreased with increasing LU concentration.

**Figure 7:**
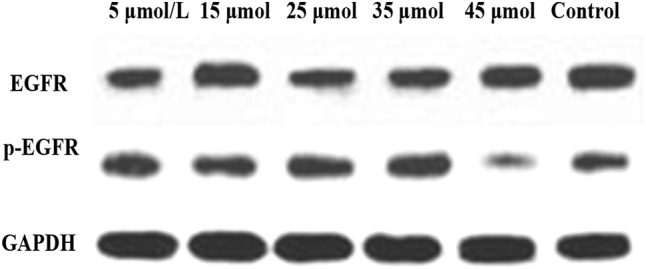
WB bands of EGFR and p-EGFR.

Figure 7 shows the WB bands of EGFR and p-EGFR. The CG exhibited the highest band intensity, followed by the 5 μmol/L LU treatment group, with intensity gradually decreasing as the LU concentration increased.

### 3.4 Akt and p-Akt protein expression

**Figure 8:**
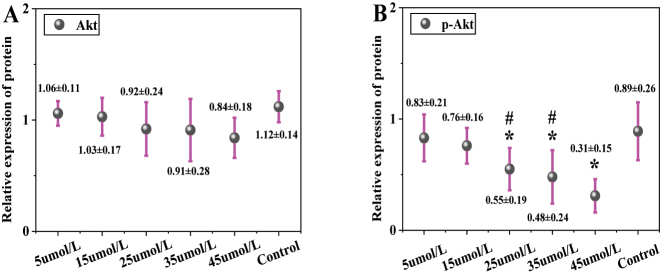
Contrast of Akt and p-Akt protein expression (A for Akt; B for p-Akt). * as against the CG, # as against 45 μmol/L LU, *P* < 0.05.

In Figure 8A, the total Akt protein expression was 1.06 ± 0.11 in the 5 μmol/L LU group, 1.03 ± 0.17 in the 15 μmol/L group, 0.92 ± 0.24 in the 25 μmol/L group, 0.91 ± 0.28 in the 35 μmol/L group, 0.84 ± 0.18 in the 45 μmol/L group, and 1.12 ± 0.14 in the CG. No significant differences in total Akt protein expression were observed between any LU concentration groups (5, 15, 25, 35, 45 μmol/L) and the CG (*P* > 0.05). In Figure 8B, the p-Akt protein expression was 0.83 ± 0.21 in the 5 μmol/L LU group, 0.76 ± 0.16 in the 15 μmol/L group, 0.55 ± 0.19 in the 25 μmol/L group, 0.48 ± 0.24 in the 35 μmol/L group, 0.31 ± 0.15 in the 45 μmol/L group, and 0.89 ± 0.26 in the CG. The p-Akt protein expression decreased significantly with increasing LU concentration. Compared to the CG, the 25 μmol/L, 35 μmol/L, and 45 μmol/L LU treatment groups showed significantly downregulated p-Akt expression (*P* < 0.05). Moreover, the p-Akt expression in the 45 μmol/L LU group was significantly lower than that in the 25 μmol/L and 35 μmol/L groups (*P* < 0.05). The p-Akt/Akt ratio was further calculated. The ratio was 0.78 ± 0.06 in the 5 μmol/L LU group, 0.74 ± 0.05 in the 15 μmol/L group, 0.60 ± 0.04 in the 25 μmol/L group, 0.53 ± 0.04 in the 35 μmol/L group, 0.37 ± 0.03 in the 45 μmol/L group, and 0.80 ± 0.06 in the CG. These results indicate that the proportion of p-Akt relative to total Akt gradually decreased with increasing LU concentration.

**Figure 9:**
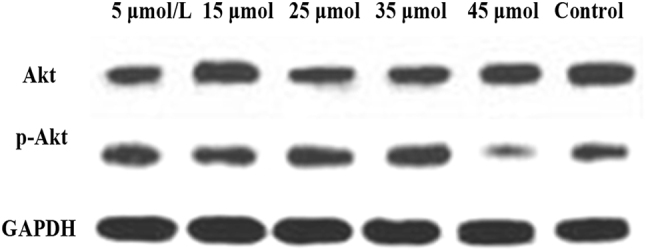
WB bands of Akt and p-Akt.

Figure 9 shows the WB bands of Akt and p-Akt. The CG exhibited the highest band intensity, followed by the 5 μmol/L LU treatment group, with intensity gradually decreasing as the LU concentration increased.

### 3.5 STAT3 and p-STAT3 protein expression

**Figure 10:**
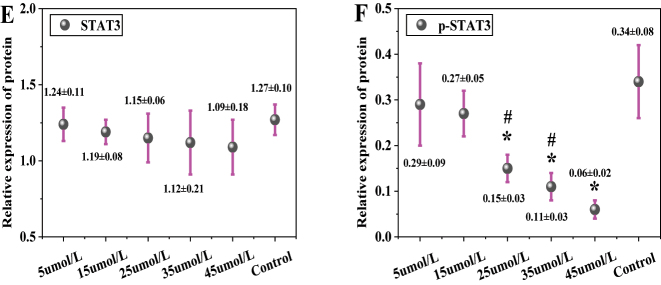
Contrast of STAT3 and p-STAT3 protein expression (A for STAT3; B for p-STAT3). * as against the CG, # as against 45 μmol/L LU, *P* < 0.05.

In Figure 10A, the total STAT3 protein expression was 1.24 ± 0.11 in the 5 μmol/L LU group, 1.19 ± 0.08 in the 15 μmol/L group, 1.15 ± 0.16 in the 25 μmol/L group, 1.12 ± 0.21 in the 35 μmol/L group, 1.09 ± 0.18 in the 45 μmol/L group, and 1.27 ± 0.10 in the CG. No significant differences in total STAT3 protein expression were observed between any LU concentration groups (5, 15, 25, 35, 45 μmol/L) and the CG (*P* > 0.05). In Figure 10B, the p-STAT3 protein expression was 0.29 ± 0.09 in the 5 μmol/L LU group, 0.27 ± 0.05 in the 15 μmol/L group, 0.15 ± 0.03 in the 25 μmol/L group, 0.11 ± 0.03 in the 35 μmol/L group, 0.06 ± 0.02 in the 45 μmol/L group, and 0.34 ± 0.08 in the CG. The p-STAT3 protein expression decreased significantly with increasing LU concentration. Compared to the CG, the 25 μmol/L, 35 μmol/L, and 45 μmol/L LU treatment groups showed significantly downregulated p-STAT3 expression (*P* < 0.05). Moreover, the p-STAT3 expression in the 45 μmol/L LU group was significantly lower than that in the 25 μmol/L and 35 μmol/L groups (*P* < 0.05). The p-STAT3/STAT3 ratio was further calculated. The ratio was 0.23 ± 0.02 in the 5 μmol/L LU group, 0.23 ± 0.02 in the 15 μmol/L group, 0.13 ± 0.01 in the 25 μmol/L group, 0.10 ± 0.01 in the 35 μmol/L group, 0.06 ± 0.01 in the 45 μmol/L group, and 0.27 ± 0.02 in the CG. The proportion of p-STAT3 relative to total STAT3 decreased significantly with increasing LU concentration.

**Figure 11:**
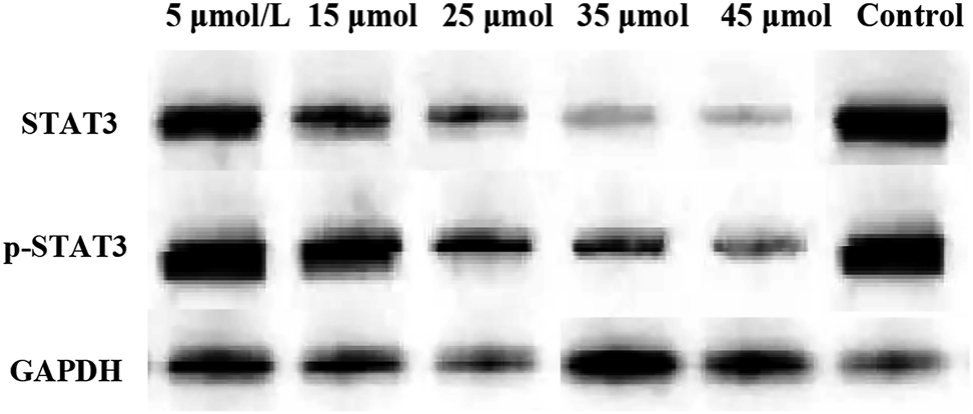
WB bands of STAT3 and p-STAT3.

Figure 11 shows the WB bands of STAT3 and p-STAT3. The CG exhibited the highest band intensity, followed by the 5 μmol/L LU treatment group, with intensity gradually decreasing as the LU concentration increased.

### 3.6 Levels of ROS

**Figure 12:**
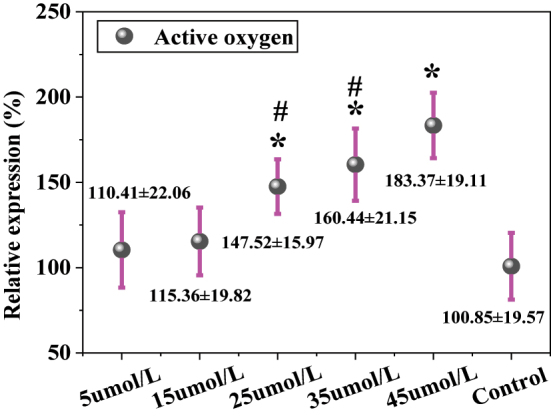
Contrast of ROS levels. * as against the CG, # as against 45 μmol/L LU, *P* < 0.05.

In Figure 12, the ROS levels were 110.41 ± 22.06 % in the 5 μmol/L LU group, 115.36 ± 19.82 % in the 15 μmol/L group, 147.52 ± 15.97 % in the 25 μmol/L group, 160.44 ± 21.15 % in the 35 μmol/L group, 183.37 ± 19.11 % in the 45 μmol/L group, and 100.85 ± 19.57 % in the CG. ROS levels increased significantly with increasing LU concentration. Compared to the CG, the 15 μmol/L, 25 μmol/L, 35 μmol/L, and 45 μmol/L LU treatment groups all exhibited significantly elevated ROS levels (*P* < 0.05), demonstrating a dose-dependent relationship.

### 3.7 Dose-response curves and kinetic parameters

Table 1 presents the kinetic parameters for LU effects.

**Table 1:** Summary of kinetic parameters for LU’s effect.

Parameter	IC50 (μmol/L)	Hill coefficient	R^2^
Proliferation inhibition	28.7 ± 1.5	1.23 ± 0.11	0.982
Apoptosis	29.4 ± 1.8	1.35 ± 0.15	0.976
ROS generation	27.8 ± 1.3	1.18 ± 0.09	0.969
p-EGFR inhibition	26.5 ± 1.2	1.19 ± 0.10	0.958
p-STAT3 inhibition	28.1 ± 1.4	1.27 ± 0.12	0.963
p-Akt inhibition	27.3 ± 1.1	1.21 ± 0.08	0.971

The inhibition curve for LU on MCF-7 cell proliferation demonstrated a good fit (R^2^ = 0.982), with an IC50 of (28.7 ± 1.5) μmol/L (95 % CI: 26.3–31.2 μmol/L), and a Hill coefficient of 1.23 ± 0.11, suggesting moderate concentration dependence (Figure 7A). At 45 μmol/L, the inhibition rate reached (72.5 ± 3.8) %, significantly higher than the 25 μmol/L and 35 μmol/L groups (*P* < 0.05).

Regarding the cell apoptosis dose-response, the AR increased in an S-shaped manner with increasing LU concentration (R^2^ = 0.976), with an IC50 of (29.4 ± 1.8) μmol/L (95 % CI: 27.1–32.0 μmol/L), and a Hill coefficient of 1.35 ± 0.15 (Figure 7B). At 45 μmol/L, the AR was (28.6 ± 2.5) %, which was 1.8 times higher than that in the 25 μmol/L group (*P* < 0.01).

Regarding the ROS level dose-response, the ROS generation curve showed a good fit (R^2^ = 0.969), with an IC50 of (27.8 ± 1.3) μmol/L (95 % CI: 25.7–30.1 μmol/L), and a Hill coefficient of 1.18 ± 0.09 (Figure 7C). At 45 μmol/L, ROS levels were 2.3 times higher than in the CG (*P* < 0.001).

Regarding the phosphorylation inhibition dose-response of signaling pathways, the IC50 values for the phosphorylation inhibition of p-EGFR, p-STAT3, and p-Akt were (26.5 ± 1.2) μmol/L, (28.1 ± 1.4) μmol/L, and (27.3 ± 1.1) μmol/L, respectively, which were consistent with the cell phenotype IC50 values (Figure 7D–F). No dose-related correlation was observed for total protein levels (*P* > 0.05).

### 3.8 Results of ROS scavenging experiment

The effects of different treatment groups on the ROS levels and apoptosis rate in MCF-7 cells are shown in Table 2. Compared with the solvent control group, the LU-alone treatment group showed a significant increase in both ROS levels and apoptosis rate (*P* < 0.05). The LU + NAC co-treatment group exhibited a significant decrease in ROS levels and apoptosis rate compared to the LU-alone group (*P* < 0.05), with no statistical difference compared to the solvent control group (*P* > 0.05). The NAC-alone treatment group had no significant effect on the cells (*P* > 0.05).

**Table 2:** Results of ROS scavenging experiment.

Experimental group	ROS level (relative to control, %)	Apoptosis rate (%)	*P*-value vs. solvent control	*P*-value vs. LU-alone group
Solvent control group	100.00 ± 8.25	5.12 ± 1.03	–	–
LU-alone group	183.37 ± 19.11	28.09 ± 4.85	<0.001	–
NAC-alone group	96.73 ± 7.58	6.05 ± 1.21	0.782	0.002
LU + NAC co-treatment group	112.64 ± 15.32	12.35 ± 2.76	0.315	<0.001

### 3.9 Results of the pathway rescue experiment

The effects of different plasmid transfections combined with LU treatment on the apoptosis rate of MCF-7 cells are shown in Table 3. Compared with the empty vector + LU group, the apoptosis rates in the EGFR-CA + LU, STAT3-CA + LU, and Akt-CA + LU groups were all significantly decreased (*P* < 0.05) and approached the level of the empty vector control group.

**Table 3:** Results of pathway rescue experiment.

Experimental group	Apoptosis rate (%)	P-value vs. empty vector control	P-value vs. empty vector + LU group
Empty vector control group	5.36 ± 1.12	–	–
Empty vector + LU group	27.83 ± 4.52	<0.001	–
EGFR-CA + LU group	13.56 ± 2.31	0.003	<0.001
STAT3-CA + LU group	14.28 ± 2.57	0.005	<0.001
Akt-CA + LU group	12.89 ± 2.14	0.002	<0.001

### 3.10 Results of LU’s effects on MDA-MB-231 cells

The results showed that LU also exerted significant, dose-dependent inhibitory effects on proliferation and pro-apoptotic effects in MDA-MB-231 cells (*P* < 0.001). Compared with MCF-7 cells, MDA-MB-231 cells treated with the same concentrations of LU exhibited a lower proliferation inhibition rate and a higher apoptosis rate. The differences were statistically significant for groups treated with LU at 25 μmol/L and higher concentrations (*P* < 0.05) (Table 4).

**Table 4:** Results of LU’s effect on MDA-MB-231 cells.

Experimental parameter	Group	Result in MDA-MB-231 cells	P-value vs. solvent control	P-value vs. MCF-7 cells at same concentration
Proliferation activity (%)	Solvent control group	99.58 ± 2.63	–	–
	5 μmol/L LU group	94.27 ± 6.85	0.531	>0.05
	15 μmol/L LU group	87.53 ± 7.21	0.176	>0.05
	25 μmol/L LU group	72.35 ± 7.81	<0.001	<0.05
	35 μmol/L LU group	63.82 ± 6.57	<0.001	<0.05
	45 μmol/L LU group	58.17 ± 6.24	<0.001	<0.01
	Positive Control group	42.36 ± 5.18	<0.001	<0.01
Apoptosis rate (%)	Solvent Control group	4.92 ± 0.87	–	–
	5 μmol/L LU group	5.73 ± 1.02	0.415	>0.05
	15 μmol/L LU group	8.26 ± 1.35	0.038	>0.05
	25 μmol/L LU group	18.63 ± 3.15	<0.001	>0.05
	35 μmol/L LU group	25.47 ± 3.89	<0.001	>0.05
	45 μmol/L LU group	32.57 ± 4.92	<0.001	<0.05
	Positive Control group	38.64 ± 5.23	<0.001	<0.01

## 4 Discussion

LU is a kind of polyphenol compound naturally occurring in some plants, especially in grape skins, red wine, and some nuts. It has a variety of biological activities and health benefits, such as antioxidant, anti-inflammatory, anticancer, and cardiovascular protection outcomes [21], [22], [23]. In general, LU is one of the hot objects in scientific research due to its various biological activities and potential health benefits, and has a broad application prospect and research value for human health [24], 25]. Therefore, it is meaningful to discuss the pathway mechanism of LU suppressing the biological characteristics of BCCs and its potential anticancer effect. In this article, MCF-7 was used as the research sample. After culture and passage, it was treated with LU solution at 5, 15, 25, 35, and 45 μmol/L, and the same amount of DMSO was added as the control. Firstly, it was found that the proliferative activity of MCF-7 decreased visibly with the increase ofLU concentration. The proliferative activity of MCF-7 at 25, 35, and 45 μmol/L LU was significantly lower as against the CG. The proliferative activity of MCF-7 at 45 μmol/L LU was significantly lower than at 25 and 35 μmol/L LU. This is similar to the research results of Li et al. (2023) [26], meaning that LU has a dose-dependent inhibitory effect on MCF-7BCCs, which can effectively suppress the multiplication of BCCs within a certain concentration range. It can be inferred that LU inhibits the proliferative activity of MCF-7BCCs in a dose-dependent manner, and this inhibitory effect may be related to the mechanism of LU in regulating cell growth signal pathway or inducing apoptosis. This study validated the mediating role of oxidative stress through ROS scavenging experiments. The ROS scavenger NAC significantly reversed LU-induced apoptosis in MCF-7 cells, indicating that LU triggers oxidative damage by promoting intracellular ROS accumulation, thereby initiating the apoptosis program. Simultaneously, pathway rescue experiments confirmed that sustained activation of the EGFR/STAT3/Akt pathway effectively blocks the pro-apoptotic effects of LU, directly demonstrating that the inhibition of this pathway is the core mechanism of LU-induced apoptosis. By integrating the dose-dependent relationship between ROS levels and apoptosis rates, as well as the causal link between pathway inhibition and apoptosis, the dual mechanisms of LU against hormone receptor-positive breast cancer were elucidated: on one hand, by specifically inhibiting the activation of the EGFR/STAT3/Akt pathway, it disrupts cell survival signaling; on the other hand, through this pathway inhibition, it indirectly promotes ROS accumulation, exacerbating oxidative stress damage. These two aspects synergistically drive cellular apoptosis.

EGFR, Akt, and STAT3 are three key signaling molecules, which play major roles in biological processes. The interaction and regulation between them are the core of many cell signaling pathways, especially in cancer research [27]. Ma et al. [28] studied the resistance of EGFR to common chemotherapeutic drugs (such as doxorubicin, daunorubicin, paclitaxel, cisplatin, and 5-fluorouracil) and ionizing radiation in BCCs, and found that the abnormally expressed EGFR kinase domain may have a major impact on the treatment resistance of BCCs. It was found that the protein expression of p-EGFR was significantly lower at 25, 35, and 45 μmol/L LU as against the CG, and significantly lower at 45 μmol/L LU as against 25 and 35 μmol/L LU. This result, combined with the study of Ma et al., can be speculated that LU has a visible inhibitory effect on the expression of p-EGFR protein, and this inhibitory effect increases with the increase of LU concentration, which may reduce the treatment resistance of BC. To further validate the role of LU in reducing therapeutic resistance in BC, subsequent combination drug experiments can be conducted: select clinically common BC drugs (*e.g.,* doxorubicin, paclitaxel), treat MCF-7 cells with each drug alone at IC_50_ or lower concentrations, and in combination with LU (at IC50 or lower concentrations). By comparing the differences in efficacy between combination therapy and monotherapy through measurements of cell proliferation inhibition rate, AR, and expression of drug resistance-related proteins (*e.g.,* ATP-binding cassette subfamily G member 2 [ABCG2]), a significant enhancement of efficacy in the combination groups would directly verify LU’s potential to enhance drug efficacy and reduce resistance. The expression of p-Akt protein was significantly lower at 25, 35, and 45 μmol/L LU as against the CG, and significantly lower at 45 μmol/L LU as against 25 and 35 μmol/L LU. The expression of p-Akt at 5, 15, 25, 35, and 45 μmol/L LU was not significantly different from that in the CG (*P* > 0.05). LU does not regulate the Akt protein by affecting its synthesis or degradation. This finding is consistent with the results reported by Kim et al. (2021) [29], who also observed that LU exerts its biological effects primarily by inhibiting the phosphorylation of key signaling proteins rather than altering total protein levels. The concentration-dependent inhibition of p-Akt by LU in this study further supports the generality of this mechanism and provides direct molecular evidence that LU exerts anti-BC effects by targeting Akt phosphorylation and subsequently modulating the EGFR/STAT3/Akt pathway. The protein expression of p-STAT3 at 25, 35, and 45 μmol/L LU was significantly lower as against the CG. This result suggests that LU may inhibit cell multiplication and promote apoptosis by significantly suppressing p-STAT. 3, rather than affecting the total STAT3 protein level. It should be noted that this study has not yet clarified the specific mechanism by which LU regulates the EGFR/STAT3/Akt pathway to mediate apoptosis in BCCs. EGFR activation is known to subsequently induce downstream STAT3 and Akt activation, and the activation of these pathways typically influences the apoptotic process by regulating the expression of apoptosis-related proteins, such as upregulating anti-apoptotic proteins to suppress apoptosis or downregulating pro-apoptotic proteins to promote cell survival. Based on the findings that LU inhibits the phosphorylation of EGFR, STAT3, and Akt, it can be speculated that LU may disrupt the regulatory balance of apoptosis-related proteins by blocking the activation of the EGFR/STAT3/Akt pathway, thereby reducing anti-apoptotic protein expression and increasing pro-apoptotic protein expression, ultimately inducing apoptosis in BCCs [30]. However, as this study did not examine the expression of key downstream apoptosis-related proteins in this pathway, the precise molecular cascade through which LU mediates apoptosis via the EGFR/STAT3/Akt pathway requires further validation in subsequent experiments, such as WB analysis of anti-apoptotic and pro-apoptotic protein expression. Although this study did not detect downstream apoptosis markers, the ROS scavenging experiment and pathway rescue experiment have clearly demonstrated that LU-induced apoptosis is dependent on the inhibition of the EGFR/STAT3/Akt pathway and ROS-mediated oxidative stress, thereby laying a foundation for further in-depth analysis of the regulatory network of apoptosis-related proteins.

This study quantitatively analyzes and, for the first time, reveals the kinetic characteristics of LU in inhibiting BCCs. The IC50 values (27–29 μmol/L) are comparable to those reported for other flavonoid compounds with anticancer efficacy, suggesting that LU exhibits clear anti-MCF-7 cell activity *in vitro*. The Hill coefficient, close to 1.0, indicates that the binding of LU to its target may involve a non-cooperative mechanism, which is consistent with the single-target inhibition mode of the EGFR/STAT3/Akt signaling pathway. Notably, the IC50 values for LU inhibition of signaling pathway phosphorylation (26.5–28.1 μmol/L) are highly consistent with the IC50 values for changes in cellular phenotypes (proliferation inhibition and apoptosis induction), confirming that LU exerts its anticancer effect by inhibiting the phosphorylation activation of the EGFR/STAT3/Akt pathway. This “upstream signal inhibition – downstream phenotype response” dose-dependent correlation provides kinetic evidence supporting the mechanism of LU action. Additionally, the dose-response curves for ROS levels and apoptosis exhibit similar Hill coefficients (1.35 vs. 1.18), suggesting that oxidative stress may play a crucial mediating role in LU-induced apoptosis. The synchronized surge in ROS generation and AR at high concentrations of LU (*e.g.,* 45 μmol/L) further supports the “oxidative damage – mitochondrial dysfunction – apoptosis execution” cascade reaction model.

The innovation of this study is mainly reflected in two aspects: First, although previous studies have confirmed the anti-breast cancer activity of LU and identified the EGFR/STAT3/Akt pathway as a key signaling axis regulating BCC proliferation and apoptosis, this study is the first to clearly demonstrate that LU targets and inhibits the phosphorylation activation of the EGFR/STAT3/Akt pathway in hormone receptor-positive MCF-7 cells, thereby downregulating the expression of downstream anti-apoptotic proteins and inducing apoptosis. This provides new target evidence for the mechanism of LU against hormone receptor-positive BC. Second, compared to previous studies that mostly focused on single targets of LU, this study systematically analyzes the compound’s overall regulatory effect on the EGFR/STAT3/Akt pathway, further refining the molecular mechanism network of LU against BC and offering more specific experimental support for the future development of LU-based adjuvant therapy strategies for hormone receptor-positive BC. This study, while clarifying the mechanism by which LU regulates proliferation and apoptosis in MCF-7 BCCs by inhibiting EGFR/STAT3/Akt pathway phosphorylation, has the following limitations. First, the research was conducted only *in vitro* using the MCF-7 cell line, a hormone receptor-positive subtype, and did not include other subtypes such as triple-negative or HER-2-overexpressing BCCs. Thus, it cannot fully represent the differential effects of LU across BC subtypes. Second, the study did not thoroughly investigate changes in the expression of specific downstream apoptosis-related molecules (*e.g.,* Bcl-2, Bax, Caspase family proteins) in the EGFR/STAT3/Akt pathway, leaving the complete molecular cascade of LU-induced apoptosis unelucidated. Third, the absence of *in vivo* animal model validation means the efficacy, metabolic characteristics, and safety of LU in the tumor microenvironment remain unclear, limiting direct data support for clinical translation. Fourth, combination therapy experiments with clinically common BC drugs (*e.g.,* paclitaxel, trastuzumab) were not conducted, preventing an assessment of LU’s potential for synergistic effects or reducing drug resistance. Based on the aforementioned limitations, future research could be conducted in the following directions: First, parallel experiments using triple-negative BCC lines (*e.g.,* MDA-MB-231) and HER-2-overexpressing cell lines (*e.g.,* SK-BR-3) could be performed to clarify the specificity of LU’s effects on different BC subtypes and its differential regulation of signaling pathways, thereby strengthening the evidence for the universality of its anti-BC actions. Second, techniques such as WB and quantitative polymerase chain reaction (qPCR) could be employed to detect the expression of apoptosis-related proteins and genes downstream of the EGFR/STAT3/Akt pathway, combined with co-immunoprecipitation to validate protein-protein interactions, thereby constructing a comprehensive molecular mechanism network linking LU, pathway molecules, and apoptotic effects. Third, an MCF-7 cell xenograft model in nude mice could be established to evaluate the *in vivo* efficacy and safety of LU by monitoring changes in tumor volume and weight after administration, analyzing the expression of phosphorylated pathway proteins in tumor tissues via immunohistochemistry, and assessing indicators such as liver and kidney function and body weight in the mice. Finally, combination therapy regimens of LU with clinical chemotherapeutic or targeted drugs could be designed, and the cell proliferation inhibition rate, AR, and expression of drug resistance-related proteins (*e.g.,* ABCG2, P-Glycoprotein [P-gp]) could be measured to explore its potential for synergistic therapy, providing an experimental basis for developing LU-based combination treatment strategies.

To further validate the subtype universality of LU’s anti-breast cancer effects, this study incorporated parallel experiments using the triple-negative breast cancer MDA-MB-231 cell line. The results demonstrated that LU also exerted significant, dose-dependent inhibitory effects on proliferation and pro-apoptotic effects in this subtype. When the LU concentration reached 25 μmol/L and above, the proliferation activity of MDA-MB-231 cells significantly decreased, and the apoptosis rate significantly increased (*P* < 0.001). This trend is highly consistent with the core effect observed in the hormone receptor-positive MCF-7 cells, confirming that the anti-breast cancer effect of LU is not limited to a single subtype but exhibits cross-subtype universality. Concurrently, subtype-specific differences were observed: under treatment with the same concentration of LU, MDA-MB-231 cells exhibited a lower proliferation inhibition rate but a higher apoptosis rate compared to MCF-7 cells. These differences were statistically significant in groups treated with 25 μmol/L and higher concentrations (*P* < 0.05). These variations may be related to the biological characteristics of triple-negative breast cancer itself, such as high invasiveness, dysregulated cell cycle control, and the activation intensity of signaling pathways. This suggests that the clinical application of LU in different breast cancer subtypes may require dosage adjustments based on subtype-specific features to achieve optimal therapeutic efficacy. These findings further broaden the application scenarios of LU in combating breast cancer, providing more comprehensive experimental evidence for its potential use as a natural compound in the personalized treatment of different breast cancer subtypes. Moreover, they lay the groundwork for subsequent in-depth exploration of the molecular differences underlying LU’s subtype-specific regulatory mechanisms in breast cancer.

## 5 Conclusions

This study investigated the effects of LU on proliferation, apoptosis, and the underlying molecular mechanisms in hormone receptor-positive MCF-7 BCCs. The results demonstrated that LU inhibited MCF-7 cell proliferation and induced apoptosis in a dose-dependent manner, while significantly increasing intracellular ROS levels. Further mechanistic studies revealed that LU specifically inhibited the phosphorylation of EGFR, STAT3, and Akt without affecting their total protein expression. ROS scavenging experiments and pathway rescue experiments confirmed that LU exerts its anti-breast cancer (BC) effects through a dual mechanism: blocking the activation of the EGFR/STAT3/Akt signaling pathway (core mechanism) and inducing intracellular ROS accumulation (mediating mechanism). Specifically, these two mechanisms work synergistically to inhibit MCF-7 cell proliferation and promote apoptosis. These findings not only fill a gap in the understanding of LU’s mechanism in hormone receptor-positive BC but also provide a new direction for the clinical development of natural compound-based adjuvant therapies for BC. Given that the EGFR/STAT3/Akt pathway is critical for BCC survival and drug resistance, LU’s targeted inhibition of this pathway may offer a potential strategy to improve treatment response and reduce drug resistance in hormone receptor-positive BC patients. Subsequent *in vivo* animal model validation and preclinical studies are warranted to further advance its clinical translation.
